# Mesocrystals from Platinum Nanocubes

**DOI:** 10.3390/nano11082122

**Published:** 2021-08-20

**Authors:** Christian Jenewein, Helmut Cölfen

**Affiliations:** Physical Chemistry, University of Konstanz, Universitätsstr. 10, D-78457 Konstanz, Germany; christian.jenewein@uni-konstanz.de

**Keywords:** mesocrystal, self-assembly, platinum, nanoparticle, nanocubes, superlattice, superstructure

## Abstract

Platinum nanoparticles are widely known for their numerous electrochemical and catalytic applications. Enhanced or novel properties that may arise when ordering such particles in a highly defined manner, however, are still subject to ongoing research, as superstructure formation on the mesoscale is still a major challenge to be overcome. In this work, we therefore established a reproducible method to fabricate micrometer-sized superstructures from platinum nanocubes. Through small-angle X-ray scattering and electron diffraction methods we demonstrate that the obtained superstructures have a high degree of ordering up to the atomic scale and, therefore, fulfill all criteria of a mesocrystal. By changing the solvent and stabilizer in which the platinum nanocubes were dispersed, we were able to control the resulting crystal habit of the mesocrystals. Aside from mesocrystal fabrication, this method can be further utilized to purify nanoparticle dispersions by recrystallization with respect to narrowing down the particle size distribution and removing contaminations.

## 1. Introduction

The fabrication of novel nanostructured materials via nanoparticle aggregation and self-assembly has proven to be one of the most promising superstructure formation pathways in the last decade [1,2,3,4,5,6]. The inherent particle-mediated nonclassical crystallization pathway of these processes has raised increasing scientific attention in more recent research [7,8,9,10,11,12,13,14]. Consequently, researchers have discovered that the behavior of a controlled aggregation and self-assembly can further be utilized to create astounding new materials with exciting properties [6,15,16,17,18,19]. In particular, colloidal methods have been demonstrated to enable access to highly ordered structures with unique functionalities [20,21,22]. Although most research in this field is focused on isotropic, spherical nanoparticles which usually self-assemble into close-packed superlattices, anisotropic nanocrystals have been shown to achieve even more complex superstructures [23,24]. While controlled self-organization of particles on the nanoscale was discovered in the 1960s, a clear and systematic description of such assemblies is often not provided [25,26]. In 2005, Cölfen and Antonietti therefore proposed a definition of colloidal crystals, which form from nonspherical crystalline building units in the form of oriented superstructures with common outer faces, and termed them mesocrystals [8]. Due to the compelling new properties of these materials, the scientific interest in mesocrystals has since increased rapidly [7,9,10,11,12,13,27]. Furthermore, mesocrystals can serve as a model system when it comes to the investigation of nonclassical crystallization processes as a result of their particle-based nature [7,11,28,29].

Based on the given IUCr definition of a “crystal”, the term “mesocrystal” itself was further specified in 2016 when Cölfen and Sturm have proposed a more distinct definition for a mesocrystal to be “a nanostructured material with a defined long-range order on the atomic scale (in at least one direction), which can be inferred from the existence of an essentially sharp wide angle diffraction pattern (with sharp Bragg peaks) together with clear evidence that the material consists of individual nanoparticle building unis” [11]. More recent work highlights the controlled self-assembly of oleic acid (OLA) stabilized iron oxide nanocubes into highly ordered arrays with lateral dimensions on the micrometer scale [30]. The detailed investigation of this process, which was carried out by Brunner et al. and Bergström et al., yielded findings that are crucial for understanding the packing arrangement and orientational ordering of cubic magnetite nanoparticles in two- and three-dimensional superlattices [31,32,33,34].

By utilizing the gas-phase diffusion technique, it is possible to synthesize up to millimeter-sized, highly crystalline mesocrystals from relatively uniform nanoparticle solutions. Therefore, it is worth investigating whether the observed assembly behavior can be transferred onto other materials and particles. Nanometer-sized platinum particles can be widely used in many applications today due to their unique catalytic and electrochemical properties [35,36]. With the rise of metamaterials, the desire for methods in order to obtain highly ordered platinum nanostructures is very compelling and therefore the first logical step towards a variety of new platinum-based materials with high surfaces and defined structures [23]. Having control over nanocrystal shape is a key factor when it comes to mesocrystal formation [7]. Hence, recent advancements in platinum nanoparticle synthesis gave access to anisotropic platinum nanocubes and various assembly strategies [37,38,39]. However, the role of different solvents or stabilizers on the formed superstructures has not yet been evaluated.

In this work, we therefore utilize the gas-phase diffusion technique to self-assemble platinum nanocubes into large, micrometer-sized mesocrystalline superstructures and explore their structures and the effect of solvent and stabilizer variation. The distinct crystal habit and the extensive amount of mesocrystals, which can be obtained from this method, allow us to observe crystallographic features from their habitus without the use of sophisticated synchrotron-based diffraction techniques. This further enables a controlled mesocrystal formation for either particle recrystallization or metamaterial fabrication without the residual dispersion contamination of solvent evaporation.

## 2. Materials and Methods

### 2.1. Chemicals

Tungsten hexacarbonyl (99%) and platinum (II) acetylacetonate (98%) were purchased from abcr. Linoleic acid (LOA) (99%), toluene (99.8+%) and oleylamine with a C-18 content of 80–90% were provided by Acros Organics (Geel, Belgium). Linolenic acid (LLA) (70%) is a TCI product (Tokyo, Japan). Oleic acid (99%) was received from Alpha Aesar (Kandel, Germany). Tetrahydrofuran (100%), hexane (98%) and ethanol (99.8+%) were purchased from VWR and Roth (Fontenay-sous-Bois, France). All chemicals were used without further purification.

### 2.2. Platinum Nanocubes

Platinum nanocubes with various fatty acids as stabilizers have been synthesized according to a slightly modified procedure, which has already been reported in the literature [37]. A mixture of 40 mg platinum (II) acetylacetonate and 3.56 g of fatty acid in 16 mL of oleylamine was placed in a two-neck Schlenk flask equipped with a condenser and attached to a Schlenk line. The mixture was heated to 120 °C under nitrogen atmosphere and vigorous stirring. After adding 100 mg of tungsten hexacarbonyl, the solution temperature was raised to 240 °C over the course of 45 min and kept at this temperature for a further 45 min under continuous stirring before cooling to room temperature again. The crude product was then separated by centrifugation at 9000 rpm for 15 min and subsequently washed with anhydrous hexane for three cycles. The final product in the form of a black oily solid was redispersed in hexane, toluene or tetrahydrofuran and stored under light exclusion.

### 2.3. 2D Self-Assemblies

Two-dimensional self-assemblies were fabricated via the solvent evaporation technique [31]. To a stable particle dispersion of diluted platinum nanocubes in tetrahydrofuran, toluene or hexane (0.5 mg/mL) the appropriate stabilizing fatty acid was added to obtain a concentration of 3 µL/mL. The substrate was placed penetrating the solvent/air interface and the solvent slowly evaporated in order to obtain self-assembled monolayers.

### 2.4. Mesocrystal Formation

Mesocrystals were synthesized by utilizing the gas-phase diffusion technique. Into a 1 mL flat bottom glass, 300 μL of a prepared particle solution containing 3 μL/mL fatty acid (99% pure) and a cleaned 5 × 7 mm silicon wafer snippet was added. The silicon snippet had been cleaned by gradual ultrasonification in ethanol, isopropanol, acetone, ethylacetate, toluene and toluene p.a. for 10–15 min each. The prepared 1 mL flat-bottom glass was then placed into a 5 mL screw cap vial containing 1.5 mL of an ethanol/solvent (50:50) mixture. The vial was then stored in a desiccator containing an ethanol-rich atmosphere for several days. The silicon snippet was carefully removed when mesocrystal formation was completed, dipped in ethanol and dried in air.

### 2.5. Analytics

Transmission electron microscopy (TEM) analysis was performed on a ZEISS LIBRA120 instrument (Carl Zeiss AG, Oberkochen, Germany) using 200 mesh carbon-coated copper grids and a 120 kV acceleration voltage. FESEM imaging was carried out on a Carl Zeiss CrossBeam 1540XB Microscope (Carl Zeiss AG, Oberkochen, Germany) equipped with a BSE detector using acceleration voltages of up to 10 kV. Samples were plasma cleaned for 60 s using a MiniFlecto from Plasma technologies (Herrenberg-Gülstein, Germany) for better imaging. To perform energy-dispersive X-ray (EDX) measurements, the microscope was equipped with an INCA X-Sight 7427 10 mm^2^ from Oxford Instruments (Abingdon, UK). SAXS measurements were conducted using a SAXS Nano Star device from Bruker (Billerica, MA, USA) equipped with a Vantec 500 detector.

## 3. Results

**Figure 1:**
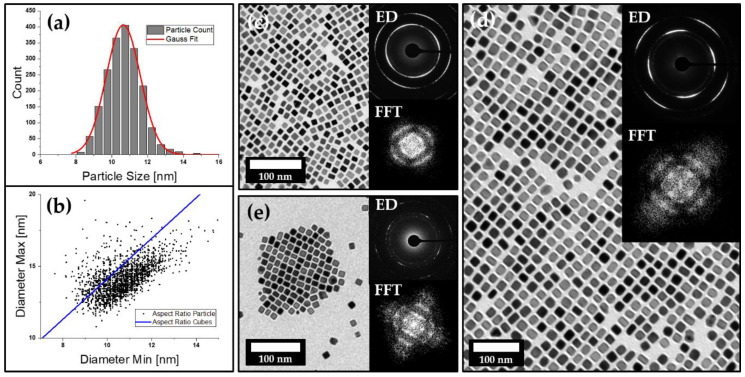
(**a**,**b**) The particle size and aspect ratio distribution of a batch of OLA-stabilized platinum nanocubes imaged by TEM (**c**) with an average size of 10.67 ± 0.98 nm. The upper inset shows an electron diffraction pattern that indicates a preferential orientation of the nanocubes on the atomic level, while the FFT (lower inset) indicates a slight ordering of the particles. TEM images (**d**,**e**) with their corresponding ED and FFT analysis further confirm these findings for LOA (**d**) and LLA (**e**) stabilized platinum nanocubes as well.

Platinum nanocubes were synthesized according to the literature in order to obtain particles with an average size of 10.7 ± 1.0 nm [37]. The reported synthesis yields a stable particle dispersion of primary cubic nanocrystals that are similar in size, shape and aspect ratio (Figure 1a–c). Particles are in situ stabilized by OLA and show a high tendency towards self-organization into two-dimensional monolayers upon solvent evaporation on the TEM sample holder. Selected area electron diffraction (SAED) analysis further displays a long-range order on the atomic scale, confirming the mesocrystalline nature of these assemblies. Besides particle size and shape, it has been observed that the stabilizer swelling, as well as the used solvents, might play a crucial role when it comes to particle self-organization [40]. To investigate if these observations are prevalent in other materials as well, we used platinum nanoparticles and modified the reported synthesis route in order to obtain various batches of platinum nanocubes coated with different stabilizers. Therefore, we chose to make use of a variety of fatty acids such as LOA and LLA as the primary stabilizer due to their chemical similarities to OLA. Unlike the common pathway of ligand exchange, the new stabilizers can be introduced through substitution of OLA in the particle synthesis route due to their chemical similarities. The obtained particle dispersions have a similar shape and size distribution and also show specific IR absorption for carbonyl vibration modes at 1645 and 1710 cm^−1^, which is exclusive to the used fatty acids, illustrating their important role in particle stabilization (Figure S1) which has already been discussed by Zhang et al. [37]. All three particle types can be successfully transferred to various organic solvents (tetrahydrofuran, toluene or hexane) to obtain a variety of particle dispersions that stay stabilized over multiple weeks when stored under exclusion of light. Analogous to what has been observed for OLA-stabilized platinum nanocubes, we also investigated the self-assembly behavior of all three types of particles. We again found a high tendency towards self-assembly into 2D mesocrystalline films upon solvent evaporation on the TEM grid (Figure 1c–e). The high degree of particle ordering can be seen in the provided TEM images, which has been verified by fast Fourier transformation (FFT). It can be observed that the 2D self-assembly behavior for various platinum nanocube batches is very similar, regardless of whether OLA, LOA or LLA has been used as the stabilizer. It is most common to observe large arrangements of particles in a primitive cubic packing; however, in some cases, they show a slight tendency towards a more hexagonal ordering. Mesocrystallinity of the obtained 2D assemblies has been further approved via SAED for all samples, as shown in Figure 1c–e inset. The SAED patterns clearly show a preferred particle orientation down to the atomic scale along their crystallographic directions, which are in correlation to the observed particle ordering. The spatial freedom of each particle—which is provided by the stabilizer shell around it—results in arcs instead of single reflexes, which can be observed in the diffraction patterns as a result of the slight particle tilting within the lattice.

**Figure 2:**
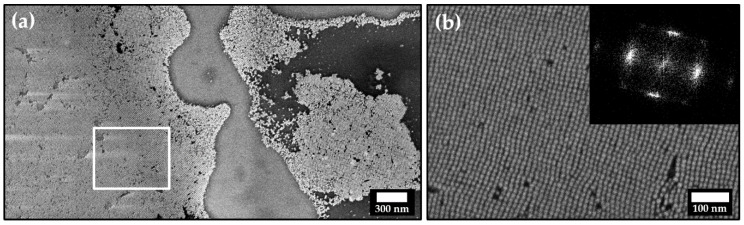
(**a**) Field emission scanning electron microscope (FESEM) image of self-assembled platinum nanocube monolayers. At higher magnification (**b**), single particles can be resolved, revealing typical crystallographic defects such as vacancies and dislocations. The inset shows the FFT pattern of image (**b**).

A much more extensive particle ordering can be achieved by slow and controlled solvent evaporation on a silicon wafer substrate, as shown in Figure 2 on the example of particle dispersion in toluene stabilized with LOA.

**Figure 3:**
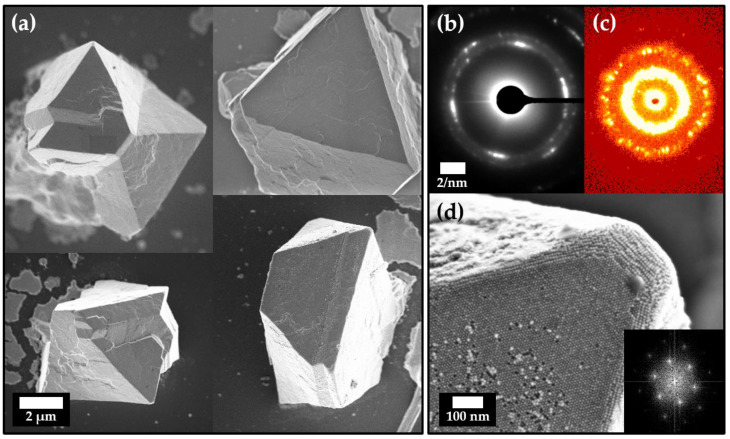
(**a**) FESEM images of various mesocrystals obtained from OLA-stabilized platinum nanocubes in hexane using the gas-phase diffusion technique with ethanol as the antisolvent. SAXS measurements Electron diffraction results (**b**) reveal a preferred crystallographic direction in which the individual particles orient. Together with SAXS measurements (**c**) of multiple mesocrystals, which show a high degree of particle ordering, it is evident that these superstructures are mesocrystalline. (**d**). The ordering on the surface appears to be hexagonal in at least one direction, which is confirmed by the FFT shown in the inset and in good agreement with previous findings by Li et al. [38].

This strong tendency towards self-assembly can also be observed when assembling particles via the so-called gas-phase diffusion technique. Due to the very slow diffusion of an antisolvent into the solvent of a stable particle dispersion, large crystals gradually form over time. Such crystals, which formed from a stable hexane particle dispersion of 1.7 mg/mL of OLA-stabilized platinum nanocubes and ethanol as the selected antisolvent, are displayed in Figure 3a. The shown superstructures emerge over the course of 14 days preferably on the polished silicon wafer substrate within the solution but can also occur on other surfaces within the particle solution, such as the glass vial for example (Figure S2). EDX measurements were performed to confirm platinum and carbon as the predominant elements within the observed superstructures. These measurements were complemented by fragmentation and subsequent transfer of the crystals onto a TEM grid to conduct SAED through the thinned corner of the superstructures (Figure S3).

In Figure 3b, the diffracted electron reflexes of such crystal fragments show a preferred crystallographic direction along which most particles within the superstructure orient themselves. This demonstrates a long-range order of atoms throughout the whole particle assembly, which is a key criterion for mesocrystals. In combination with the analysis obtained from small-angle X-ray scattering (SAXS) measurements, we can further prove that we were able to obtain platinum-nanocube-based mesocrystals. Evaluation of the SAXS data displays an average particle periodicity of 10.8 ± 3.2 nm over a multitude of scanned mesocrystals, which is in good accordance with the particle size of our samples (Figure S4). Due to the small size of the mesocrystals, it is not possible to investigate the exact crystallographic structure with the available beam size of a standard X-ray source. Nevertheless, the majority of the mesocrystals show a trigonal habitus with a size of roughly 10–20 µm. In addition, the apparent hexagonal particle ordering on the mesocrystal surface (Figure 3d) strongly suggests an underlying hexagonal crystal system. These observations are in good agreement with the obtuse rhombohedral superlattice structure, which has been found by Li et al. for platinum nanocube self-assemblies formed from solvent evaporation [38]. This suggests that both assembly pathways follow a similar nonclassical crystallization process yet their assembling strategies are quite different. However, extensive analytical effort in form of synchrotron-based wide- and small-angle diffraction would be needed in order to clarify if they exhibit exactly the same crystallographic structure.

As mentioned before, the gas-phase diffusion process of ethanol into hexane to form the mesocrystals is a very slow process, which is why mesocrystal formation takes place over the course of several weeks. By reducing the time scale at which mesocrystals form, we want to investigate a faster formation route and explore how this will impact the mesocrystal morphology to learn more about the formation of these crystals. The key advantage of the gas-phase diffusion technique is that mesocrystals can be obtained from various organic solvents as long as the nanoparticle dispersions are stable in the chosen solvent. When choosing a slightly more polar substance as the primary solvent, a smaller amount of antisolvent is needed in order to destabilize the particle dispersion, which can reduce mesocrystal formation time from 14+ days (hexane) down to 24 h (tetrahydrofuran). Thus, we investigated mesocrystal formation of platinum nanoparticles from three different solvents: hexane, toluene and tetrahydrofuran. In addition, we also examined crystal formation from particles stabilized with three different fatty acids: OLA, LOA and LLA. In all nine cases, we managed to obtain micrometer-sized mesocrystals of larger quantities. Table 1 gives an overview of crystallization times and the resulting shape of the obtained crystals.

**Figure 4:**
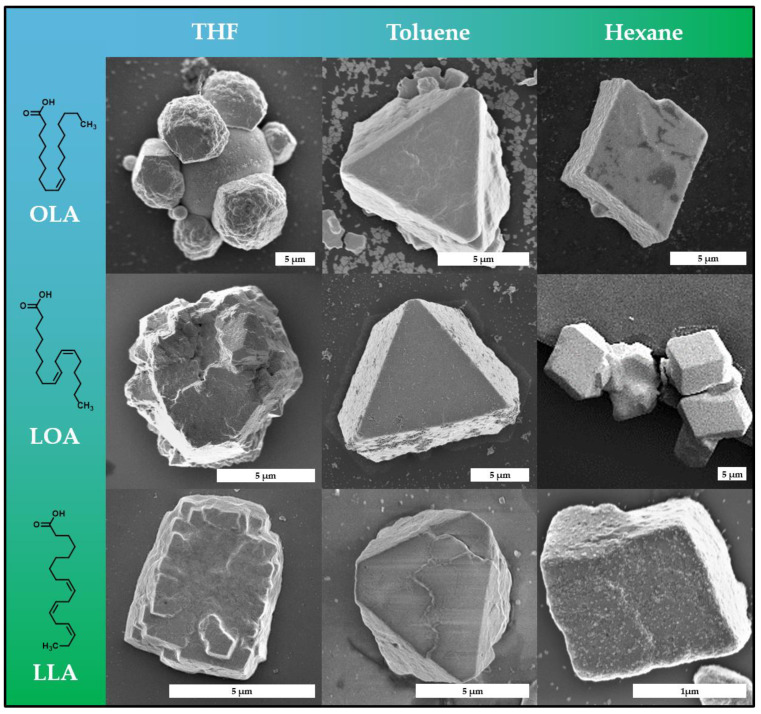
A library of representative mesocrystals obtained from platinum nanocubes stabilized by three different fatty acids (OLA, LOA and LLA) in three different organic solvents (tetrahydrofuran, toluene and hexane). Mesocrystal shape appears to be influenced mainly by the solvent rather than the stabilizer, as mesocrystals crystallized from hexane exhibit a hexagonal shape, mesocrystals from toluene exhibit a truncated trigonal pyramidal shape and mesocrystals from THF exhibit an undefined polycrystalline shape.

Observations across multiple sample batches reveal that platinum mesocrystals grown from particle dispersions in either hexane or toluene emerge into highly defined crystals with smooth surfaces and distinct habitus, whereas mesocrystals from THF feature less determined structures (Figure 4). A more detailed investigation suggests that crystals grown from hexane primarily result in rhombohedral-shaped crystals, which is again further evidence related to the hexagonal packing of the particles observed earlier. This is in further agreement with the results of a similar system, reported in the literature [38]. Mesocrystals obtained from toluene, on the other hand, predominantly exhibit a trigonal truncated pyramid habitus. The reason for this can be seen in the different swelling of the stabilizer layer on the crystal surface, which changes the external shape of the nanoparticle and thus the packing possibilities. The mesocrystal formation in THF, however, results primarily in undefined crystals with a significant amount of observable defects. These defects are most prevalent in form of crystal twinning and grain boundaries, as seen in Figure 4 and Figure S5. Platinum nanocube mesocrystals crystallized from THF can therefore be considered as mostly polycrystalline in the sense that they exhibit multiple mesocrystalline domains. We assume that this is a result of the accelerated destabilization of the particle dispersion via the gas-phase diffusion when using THF. In addition to the high-resolution SEM imaging of the mesocrystal surface, it is indicated that the hexagonally packed platinum nanoparticle planes stack in a face-centered cubic manner when observed at these grain boundaries. However, advanced synchrotron-based SAXS and WAXS analytics would be needed to exactly clarify the mesocrystalline structure of such small crystals, as stated earlier [38]. It is noteworthy to mention that various other crystals with a different shape can also be found in each sample; however, the majority of the crystals were identified as the above stated. While the chosen solvent appears to have a substantial impact on the mesocrystal formation process as discussed, we also managed to utilize a variety of different nanoparticle stabilizers while maintaining micrometer-scale mesocrystal formation. It could be shown that it is possible to obtain mesocrystals from all three stabilizers (OLA, LOA and LLA) in all three solvents (hexane, toluene and tetrahydrofuran). Although all three stabilizers are very similar regarding their chemical composition, they exhibit essential physical differences such as solubility or melting temperature. The latter is a very important feature when it comes to future investigation on the role of temperature during mesocrystal formation. The much lower melting point of LLA (−11 °C) in comparison to OLA (+16 °C), for example, enables a much broader temperature range at which nonclassical crystallization can be conducted and examined.

**Figure 5:**
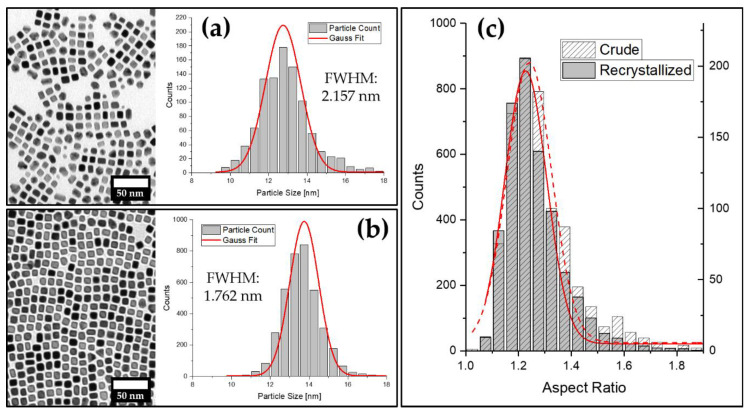
(**a**) TEM image of a crude batch of platinum nanocubes with the corresponding particle size distribution; (**b**) TEM image and corresponding particle size distribution of the same batch as shown in (**a**) after recrystallization from THF within 24 h; (**c**) comparison of particle aspect ratio distributions of both samples: crude (**a**) and recrystallized (**b**).

So far, we were able to demonstrate the feasibility of the gas-phase diffusion technique to synthesize platinum-nanocube-based mesocrystals analogous to iron oxide nanocubes. In a complementary study, our group further reported on the utilization of this method for recrystallization of a stable particle dispersion to narrow down its size distribution [41]. Similar to recrystallization, as it is commonly used in organic and inorganic preparative chemistry, a batch of crude nanoparticles can slowly crystallize from solution as described above. Subsequent to the removal of the supernatant solution, the remaining crystals can be redispersed in compatible organic solvents to obtain a recrystallized particle sample. We therefore investigated the applicability of this method for the herein described platinum-nanocube-based mesocrystals. Figure 5a shows examples of crude and a recrystallized samples of LOA-stabilized platinum nanocube particle dispersion in tetrahydrofuran. After one recrystallization cycle of 24 h, the polydispersity of the particle solution decreased notably (Figure 5b), which is indicated by a 19% reduction in the full width at half maximum (FWHM) of the Gaussian fit from 2.16 to 1.76 nm. Additionally, the evaluation of the aspect ratios of the recrystallized particles indicates the removal of the cuboid and rod-shaped particles, which can be seen in the provided TEM images and the measured aspect ratios illustrated in Figure 5c.

## 4. Conclusions

The strong tendency towards self-assembly of fatty-acid-stabilized platinum nanocubes was utilized in this work to establish a gas-phase diffusion synthesis route to assemble platinum nanocubes into highly ordered superstructures. These superstructures in form of micrometer-sized crystals were subsequently identified as mesocrystals by the means of SEM, SAXS and ED due to their long-range particle ordering on the atomic scale. By controlling various parameters such as particle concentration, diffusing agent, solvent type and crystallization time, we were able to find the conditions under which the nonclassical crystallization process reproducibly yields large quantities of platinum-nanocube-based mesocrystals. We also highlighted how this technique allows the fabrication of platinum mesocrystals from either different organic solvents or variable stabilizers in the form of three different fatty acids. Furthermore, we verified our previous observations that this method can be used to purify a platinum particle dispersion by narrowing down the particle size distribution through recrystallization from mesocrystals. In addition, the observed crystallinity and crystal habits of the platinum-nanocube-based mesocrystals appear to be driven by the properties of the used particles and the solvent they are dispersed in. Although we were able to determine the conditions under which the platinum mesocrystals form, there are still several factors that play a major role in mesocrystal formation and are yet to be understood. Among them are nanoparticle shape, stabilizer swelling and the solvation shell in different solvents, which can have a significant consequence on the packing and morphology of the mesocrystal [40]. Our results outline that these factors are relevant for platinum-nanocube-based mesocrystals. By demonstrating the ability to obtain comparable mesocrystals from building blocks stabilized through various surfactants, we provide a practical toolkit that can be used for a broad investigation of fundamental nonclassical crystallization processes at different temperatures.

## Figures and Tables

**Table 1:** Crystallization times for mesocrystals from platinum nanocubes in various dispersion solvents and their resulting predominant crystal shape.

Dispersion Solvent	Crystallization Time	Predominant Crystal Shape
tetrahydrofuran	1 day	polycrystalline
toluene	7–14 days	trigonal truncated pyramid
hexane	14–28 days	rhombohedral

## Data Availability

Not applicable.

## Supplementary Materials

The following are available online at https://www.mdpi.com/article/10.3390/nano11082122/s1, Figure S1: IR absorption spectra of fatty-acid-stabilized platinum nanocubes. Figure S2: Platinum mesocrystals on quartz surfaces, Figure S3: Elemental analysis of platinum mesocrystals, Figure S4: Small-angle X-ray scattering of platinum mesocrystals, Figure S5: Polycrystalline platinum mesocrystals.
