# Supplementary material for: Mesocrystals from Platinum Nanocubes
Source: Nanomaterials (Basel). 2021 Aug 20;11(8):2122. doi: 10.3390/nano11082122 (PMC8398057; doi:10.3390/nano11082122)
Supplement: Supplementary file 1 [file nanomaterials-11-02122-s001.zip › nanomaterials-1319824-supplementary.pdf]

Supplementary Materials

Mesocrystals from Platinum Nanocubes

Christian Jenewein [1] and Helmut Cölfen [1,*]

[1] Physical Chemistry, University of Konstanz, Universitätsstr. 10, D-78457 Konstanz
* Correspondence: helmut.coelfen@uni-konstanz.de; Tel.: +49-7531-88-4063

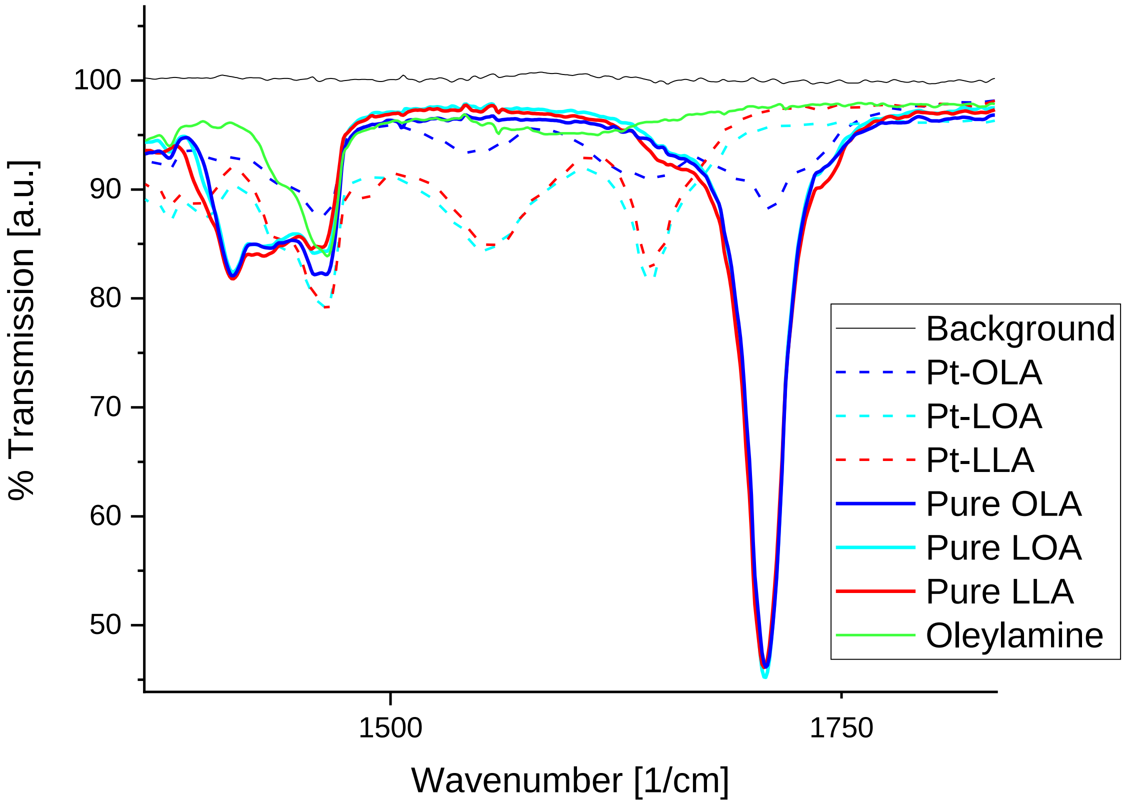

Figure S1: IR Absorption Spectra of Fatty Acid Stabilized Platinum Nanocubes. ATR-IR spectra of platinum nanocubes stabilized by OLA, LOA and LLA in comparison to reference spectra of pure OLA, LOA and LLA as well as Oleylamine which is also used for particle synthesis. Shown are the typical wavenumbers for carbonyl vibration modes. Shifted carbonyl modes are due to the binding on the platinum particle surfaces.

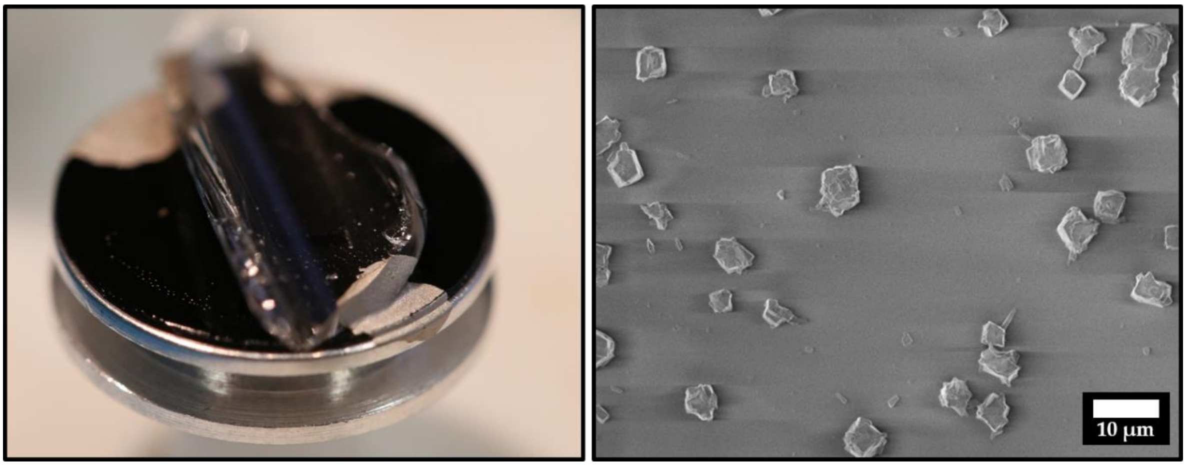

Figure S2: Platinum Mesocrystals on Quartz Surfaces. OLA stabilized Pt Nanocube mesocrystals formed on a quartz glass surface (left image) shown in FESEM image (right image). The formation of multiple micrometer sized superstructures on the quartz glass surface can be observed in in this and all the other samples.

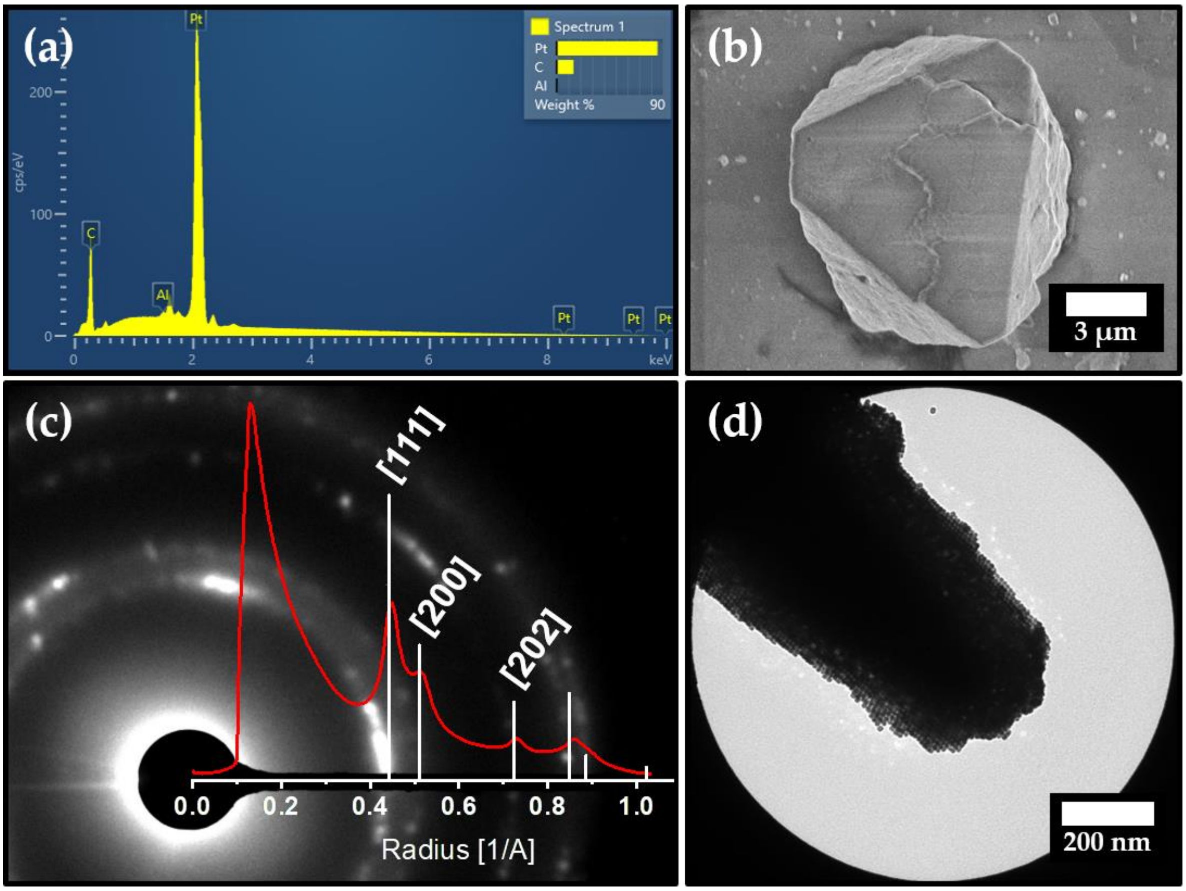

Figure S3: Elemental Analysis of Platinum Mesocrystals. Upper images show point EDX analysis (a) of the OLA stabilized platinum nanocube based mesocrystal imaged by SEM (b). Image (c) illustrates the integrated radial intensity of the diffraction signals of the SAED in Figure 3b with the corresponding platinum [hkl] references of the fragmented platinum mesocrystal piece shown in TEM image (d)

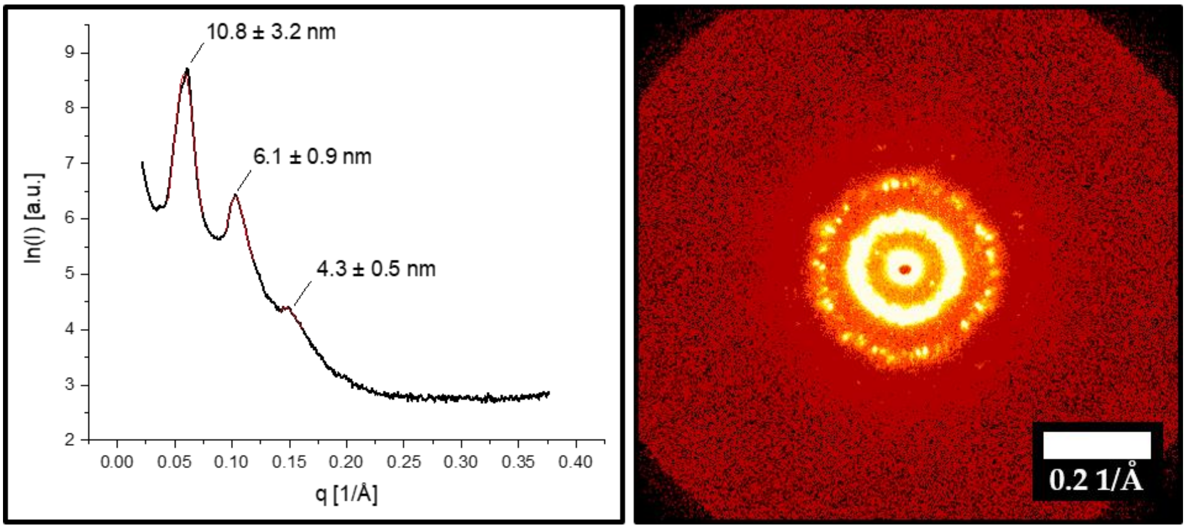

Figure S4: Small Angle X-Ray Scattering of Platinum Mesocrystals. SAXS analysis of a multitude of OLA stabilized platinum nanocube mesocrystals formed from hexane. Three main signals have been identified and corrected via a Guassian peak fit to determine the peak center and its width. The main signal at 10.8 ± 3.2 nm is in good accordance with the determined particle size (10.8 ± 3.2 nm) of the used particles. Signals at 6.1 ± 0.9 nm and 4.3 ± 0.5 nm most likely correspond to diffraction signals of higher miller indices which cannot be properly determined without the use of synchrotron based SAXS from various angles.

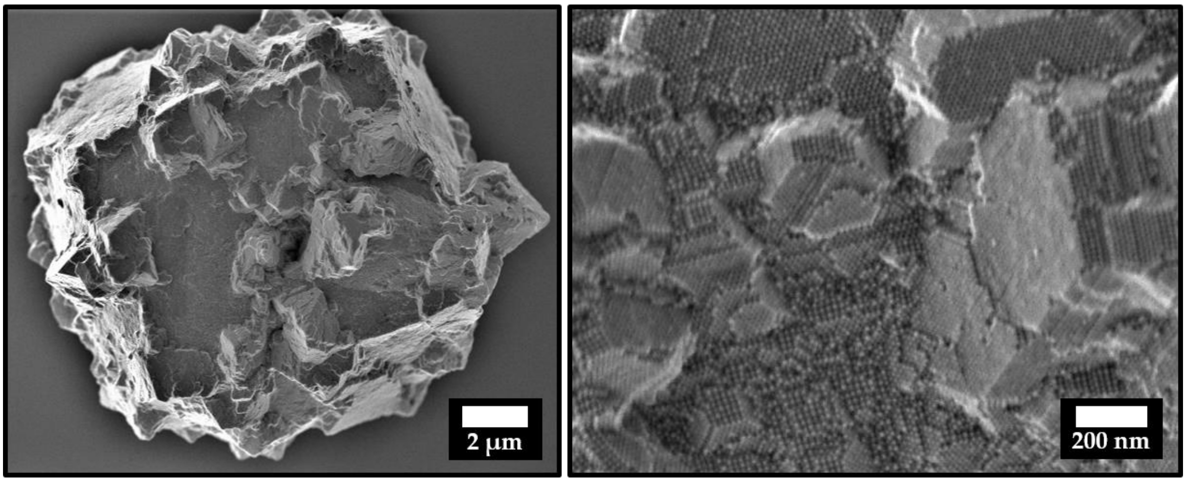

Figure S5: Polycrystalline Platinum Mesocrystals. FESEM images of polycrystalline LOA stabilized platinum nanocube based mesocrystals crystallized from THF over the course of 24 hours. On the left image, a mesocrystal with various twinning defects is shown. The right image highlights the polycrystalline character of the mesocrystal, showing various mesocrystalline domains in the crystal which are oriented in a different direction, revealing a cubic stacking of the hexagonally ordered planes of particle arrays.
